# Thyroid lesions of neuroendocrine origin? Thinking of a “polka‐dotted” zebra! Case series from three Italian referral centers and review of the literature

**DOI:** 10.1111/jne.70061

**Published:** 2025-06-21

**Authors:** Tiziana Feola, Alessia Cozzolino, Federica Grillo, Maria Francesca Birtolo, Irene Aini, Erika Messina, Roberto Minotta, Alessia Filice, Isabella Zanata, Paola Razzore, Manila Rubino, Andrea M. Isidori, Annamaria Colao, Antongiulio Faggiano, Elisa Giannetta, NIKE group, Irene Aini, Manuela Albertelli, Ylenia Alessi, Simone Antonini, Luigi Barrea, Elio Benevento, Maria Francesca Birtolo, Federica Campolo, Celeste Cantone, Silvia Carra, Rob‐erta Centello, Alessia Cozzolino, Federica De Cicco, Valentina Di Vito, Beatrice Fazzalari, Stefano Gay, Elisa Giannetta, Federica Grillo, Elia Guadagno, Valentina Guarnotta, Iderina Hasballa, Alice Laffi, Andrea Lania, Alessia Liccardi, Rossella Mazzilli, Erika Messina, Roberto Minotta, Giovanna Muscogiuri, Carla Pandozzi, Gabriella Pugliese, Giulia Puliani, Alberto Ragni, Manila Rubino, Rosaria Maddalena Ruggeri, Franz Sesti, Maria Grazia Tarsitano, Ludovica Verde, Giovanni Vitale, Virginia Zamponi, Isabella Zanata, Roberta Modica, Giuseppe Fanciulli, Anna La Salvia, Erika Maria Grossrubatscher, Francesco Ferrau, Alessandro Veresani, Flaminia Russo

**Affiliations:** ^1^ Department of Experimental Medicine “Sapienza” University of Rome Rome Italy; ^2^ Department of Neuroendocrinology Neuromed Institute, IRCCS Pozzilli Italy; ^3^ Anatomic Pathology Unit University of Genova and Policlinico San Martino Hospital Genoa Italy; ^4^ San Martino Polyclinic Hospital Genova Italy; ^5^ Endocrinology, Diabetology and Medical Andrology Unit IRCCS Humanitas Research Hospital Milan Italy; ^6^ Department of Biomedical Sciences Humanitas University Milan Italy; ^7^ Endocrinology Unit Azienda Ospedaliera Universitaria Sassari Sassari Italy; ^8^ Department of Clinical and Biological Sciences, Internal Medicine San Luigi Gonzaga Hospital, University of Turin Orbassano Italy; ^9^ Department of Clinical Medicine and Surgery, Endocrinology, Diabetology and Andrology Unit Federico II University of Naples Naples Italy; ^10^ Department of Medical Sciences, Section of Endocrinology and Internal Medicine University of Ferrara Ferrara Italy; ^11^ Endocrinology Unit Mauriziano Hospital Turin Italy; ^12^ Clinic for Endocrinology and Diabetology Ente Ospedaliero Cantonale (EOC) Bellinzona Switzerland; ^13^ Division of Gastrointestinal Medical Oncology and Neuroendocrine Tumors IEO, European Institute of Oncology, IRCCS Milan Italy; ^14^ Centre for Rare Diseases (ENDO‐ERN accredited), Policlinico Umberto I Rome Italy; ^15^ Endocrinology Unit, Department of Clinical and Molecular Medicine Sant'Andrea Hospital, ENETS Center of Excellence, Sapienza University of Rome Rome Italy

**Keywords:** medullary thyroid carcinoma, neuroendocrine neoplasms, neuronendocrine tumors, thyroid metastases, thyroid tumors

## Abstract

**Background:**

Neuroendocrine neoplasms (NENs) may metastasize very rarely to the thyroid. The current paper aims at identifying peculiar thyroid nodule's features that could prompt their diagnosis and analyzing therapeutic approach and patient's outcome.

**Materials and Methods:**

A case series of three patients have been collected from three Italian referral centers. Moreover, we performed a keyword based PUBMED search, using relevant keywords.

**Results:**

We included in the review 27 papers and 33 cases have been identified. Patients’ age ranged from 17 to 85 years (mean age: 55.8 ± 14.2 years), 14 males, 42.4%. The majority of cases (48.5%) originated from a thoracic NEN. Median time to diagnosis from the primary tumor was 48 months (range 1–252 months). At ultrasound, they were generally hypoechoic nodules with irregular margins. The diagnosis was made by fine‐niddle aspiration in the majority of cases, followed by nuclear medicine imaging. At immunohistochemistry, chromogranin A and synaptophysin were expressed in almost all of them, with negative calcitonin and thyroid transcription factor‐1. Surgery or systemic treatment were needed according to primary tumor, disease stage, and patients’ general condition. Prognosis was variable, better if primary tumor origin was thoracic.

**Conclusions:**

Thyroid metastases from NENs should be considered in the diagnostic work‐up of suspicious thyroid nodules in patients with a positive medical history of previous NEN, mainly of thoracic origin. Immunohistochemistry is the key diagnostic tool for their identification. A prompt and correct diagnosis is mandatory because of its crucial prognostic and therapeutic implications.

## Graphical abstract

Thyroid metastases from neuroendocrine neoplasm (NEN) should be considered in case of suspicious thyroid nodules in patients with a positive medical history of NEN, mainly of thoracic origin, even after many years from the initial diagnosis. The differential diagnosis from primary intrathyroidal NEN is challenging.

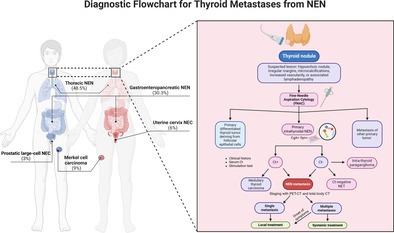

## 1 INTRODUCTION

Neuroendocrine neoplasms (NENs) are a heterogeneous group of malignancies arising from neuroendocrine cells, found in various organs and tissues, with varying degrees of aggressiveness and clinical behavior.^1^ NENs rarely metastatize to the thyroid gland, accounting for only 1.0%–4% of cases; when they do occur, they often mimic primary thyroid tumors both clinically and histologically.^2^ This can make diagnosis particularly challenging, especially in patients without a known history of malignancy elsewhere.

Indeed, endocrine clinical practitioners should consider the thyroid gland as a potential site of NEN metastasis, which have to be differentiated from more common thyroid entities: (1) primary differentiated thyroid tumors deriving from follicular epithelial cells; (2) primary intrathyroidal NENs, such as medullary thyroid carcinomas (MTCs), calcitonin (Ct)‐negative neuroendocrine tumors (CNNETs), and intrathyroidal paragangliomas; and (3) metastasis of other tumors that more commonly spread to the thyroid.^3^

MTCs, originating from parafollicular cells (or C‐cells), account for 5%–8% of all thyroid cancers and exhibit a distinctive immunohistochemical profile. MTCs typically express Ct, carcinoembryonic antigen (CEA), thyroid transcription factor 1 (TTF1), and other neuroendocrine markers, including chromogranin A (CgA) and synaptophysin (Syn). Futhermore, Ct is highly sensitive and specific circulating marker for MTC. Normal serum Ct levels and negative immunohistochemistry for Ct generally rule out MTC in cases of intrathyroidal NEN. However, elevated Ct levels are not exclusive to MTC, as Ct can be produced by extrathyroidal NENs, particularly foregut‐derived gastroenteropancreatic (GEP)‐NETs.^4^, ^5^, ^6^, ^7^, ^8^

Other rare differential diagnoses, include CNNET,^9^ and intrathyroidal paraganglioma, arising from the inferior laryngeal paraganglia and representing the 0.012% of head–neck tumors.^10^ Diagnosing these conditions is particularly challenging due to their histological similarities with MTC, follicular neoplasms, and intrathyroidal parathyroid adenomas.

Given the rarity of these entities, the current paper shows a case series of three patients with thyroid metastases from NENs, accompanied by a literature review. The study aims to determine the types of NENs most frequently metastasizing to the thyroid, highlight the differential diagnosis challenges between metastatic and primary intrathyroidal NENs—particularly MTC—and identify specific thyroid nodule characteristics that could aid in diagnosis. Furthermore, the paper analyzes therapeutic approaches and patient outcomes to enhance the clinical management of this rare condition.

## 2 MATERIALS AND METHODS

A case series of three patients with a confirmed cytological/histological diagnosis of thyroid metastasis from NENs have been collected from three Italian referral centers, participating in the ‘NIKE’ project (Neuroendocrine tumors Innovation Knowledge and Education). Written informed consent was obtained from the individuals for the publication of any potentially identifiable images or data included in this article.

Moreover, we performed a keyword based PUBMED search, using relevant keywords [(thyroid metastases and neuroendocrine tumor) OR (thyroid metastases and neuroendocrine neoplasm) OR (thyroid and metastasis and carcinoid)]. The search was last updated in October 2024, and only English language studies were considered. Titles and abstracts have been screened for articles selection, identifying only those that dealt with thyroid metastasis from NENs. The selected abstracts were further assessed for a full‐text evaluation. Finally, 27 papers were considered eligible and were included in the review. Overall, 33 cases have been identified. Data regarding year of publication, thyroid metastasis and primary tumor's characteristics, time from NEN diagnosis, modalities of metastasis' detection, treatment of primary and metastasis, and last follow‐up have been extracted.

## 3 CASE SERIES

Details of case series are summarized in Table 1.

**TABLE 1:** Details of case series on thyroid metastasis from NEN in three Italian referral centers.

Referral center	Age, sex	Site of thyroid MTS	US features	Site of primary (cm)	Thyroid MTS (cm)	MTS diagnosis (timing from primary)	Primary histology, Ki 67	Thyroid MTS, immunohistochemistry, Ki 67, Limph nodes status	Serum NE biomarkers	Treatment of primary	Treatment of thyroid MTS	Follow‐up
Case 1	53, M	Right lobe	2 nodules, the largest solid, hypoechoic with irregular margin + three enlarged lymph nodes	Pancreas (2.0)	1.0 (largest nodule)	Octreoscan + FNA (3 months)	Pancreatic NET, Ki‐67 15%	Multiple localization of NET, CgA+, Syn+, TTF1−, CDX2−, CK19−, CK7−, Ct−, Ki 67 < 1% + massive nodes MTS	CgA 190 U/L (0–34)	Cephaloduodenopancreatectomy and peripancreatic lymphadenectomy	Right hemithyroidectomy with cervical lymph nodes dissection	PD (liver) SSA, INFa, Cisplatin +Etoposide
Case 2	61, F	Right lobe	Hypoechoic with regular margins and peripheral vascularity + suspicious lymph nodes	Right Ovary (3.3 × 3.2 × 2.4)	0.8 × 0.5 × 0.9	^68^Ga‐DOTATOC PET/CT + FNA (360 months)	Cystic teratoma with area of struma ovarii, including focus of NET, Ki 67 <2%	NET, Syn+, galectin+/−, TTF1+/−, CDX2+/−, Tg+/−, CgA−, CT−, Ki 67 <2% + limph nodes (3/5) MTS	CgA and 5HIAA normal, Ct <2 pg/ml	Right oophorectomy, removal of bulky lymph nodes and peritoneal washing	Total thyroidectomy and lymph nodes dissection	PD (liver) SSA
Case 3	74, M	Right lobe	Solid, hypoechoic with regular margins, internal macrocalcifications and peripheral vascularity	Lung (3.0 × 2.1)	2.1 × 2.0 × 2.2	FNA+ ^18^F‐FDG PET/CT (prior of the primary)	Small‐cell lung neuroendocrine carcinoma, Ki67 90%	Poorly differentiated neoplastic cells, CgA+, Syn+, CAM 5.2+, INSM1+, TTF1−, Ki 67 NA^a^	NSE 11.1 μg/L (< 10.0) Ct <1 pg/ml	NA	NA	NA

Abbreviations: +/−, focally expressed; 5‐hydroxy‐indole acetic acid, 5‐HIAA; CAM5.2, pancytokeratin; CDX2, caudal‐related homeobox transcription factor 2; CD56, cluster of differentiation 56; CEA, carcinoembryonic antigen; CgA, chromogranin A; CK7, cytokeratin 7; CK19, cytokeratin 19; Ct, calcitonin; FNA, fine needle aspiration; INSM1, Insulinoma‐associated protein 1; MTS, metastasis; NA, not available; NE, neuroendocrine; NSE, neuron specific enolase; NET, neuroendocrine tumor; PAX8, paired box protein 8; PD, progressive disease; PET/CT, positron emission tomography‐computed tomography; Syn, synaptophysin; SSA, somatostatin analogs; Tg, Thyroglobulin; TTF1, thyroid transcription factor 1; US, ultrasound.

^a^ Cytological evaluation.

### 3.1 Case 1

In April 2000, a 53‐year‐old man with a history of hypertension was admitted to Niguarda Hospital (Milan). Physical exam and labs were normal, except for elevated CgA (190 U/L with a normal range of 0–34 U/L).

Abdominal computed tomography (CT) revealed a 2 cm enhancing mass in the pancreatic head, liver lesions, and enlarged lymph nodes. 111In‐pentetreotide scintigraphy confirmed uptake in the pancreas and liver, and moderate uptake in the right side of the neck was also detected.

**FIGURE 1:**
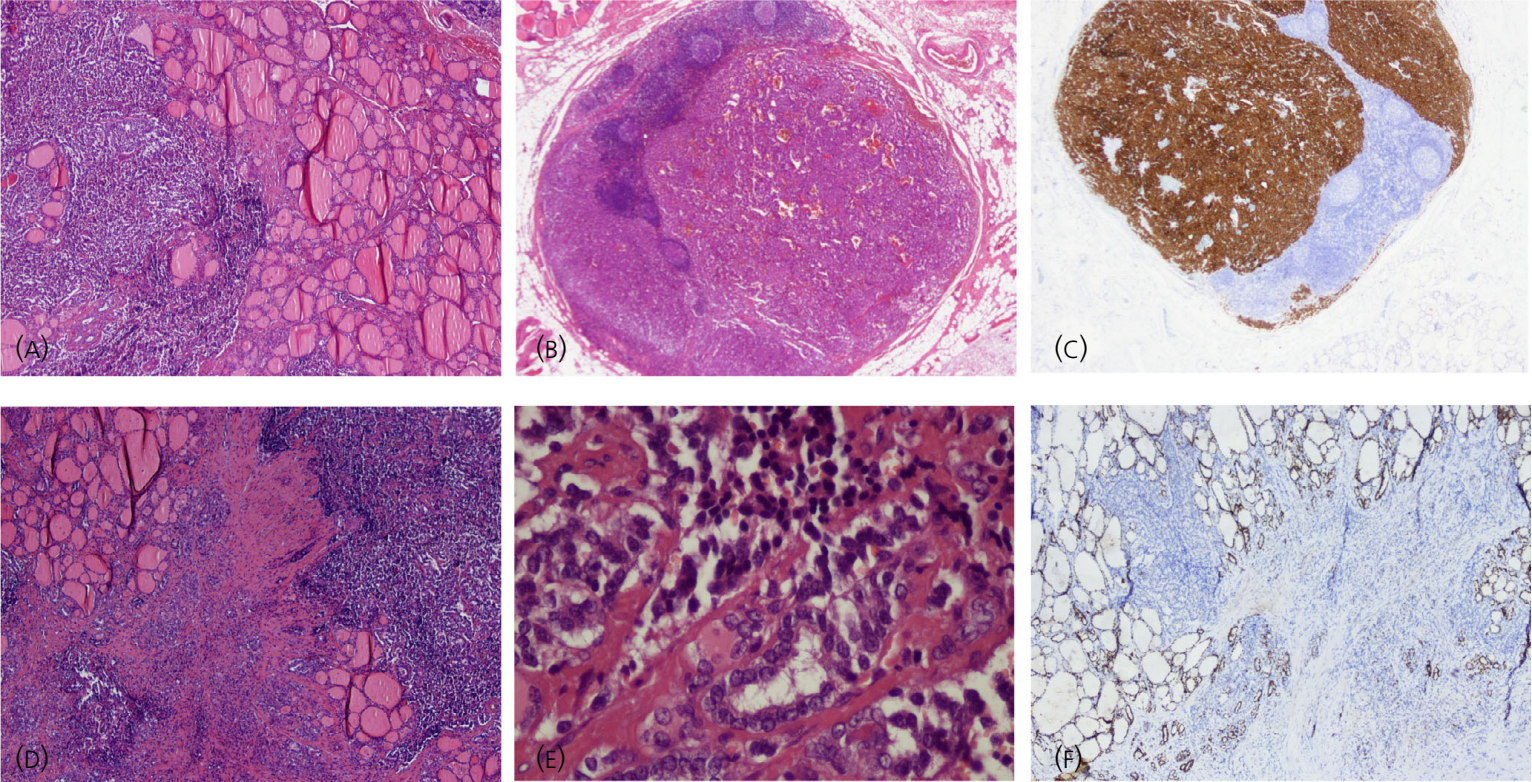
(A) Thyroid metastasis of neuroendocrine tumor. Hematoxylin–eosin staining (20×); (B) Lymph node metastasis of neuroendocrine tumor. Hematoxylin–eosin staining (20×); (C) Lymph node metastasis of neuroendocrine tumor. Hematoxylin–eosin staining. Immunohistochemistry for synaptophysin (20×): Diffuse cytoplasmic expression in neuroendocrine cells; (D) Metastatic foci of neuroendocrine tumor in close relationship with an area of papillary carcinoma of the thyroid. Hematoxylin–eosin staining (20×); (E) Metastatic foci of neuroendocrine tumor in close relationship with an area of papillary carcinoma of the thyroid. Hematoxylin–eosin staining (400×); (F) Metastatic foci of neuroendocrine tumor in close relationship with an area of papillary carcinoma of the thyroid. Hematoxylin–eosin staining. Immunohistochemistry for TTF1 (20×): Strong nuclear staining in thyroid and papillary carcinoma.

An exploratory laparotomy confirmed the neuroendocrine origin. Hence, cephaloduodenopancreatectomy and liver resection (segments VI, VII, and VIII) were performed. Histopathological findings confirmed the diagnosis of pancreatic well‐differentiated neuroendocrine carcinoma (according to World Health Organization WHO 2000 classification) with hepatic and lymph nodal metastasis; at immunohistochemistry the tumor cells were strongly positive for synaptophysin and CgA, and a MIB‐1 proliferative index of 15% was found. Three months later, CgA increased, though imaging was negative. Therefore, a total body 111In‐pentetreotide scintigraphy was performed, and three areas of intense uptake projecting to the right side of the jugulous and another in the right submandibular latero‐cervical area were detected. The subsequent neck US revealed at least three enlarged lymph nodes with pathological echo‐structural characteristics and two nodular formations in the right lobe of thyroid, the largest with a diameter of 1.0 cm, hypoechoic appearance, and blurred and poorly defined margins. The fine‐needle aspiration (FNA) of this nodule showed neoplastic cells and led to right hemithyroidectomy with cervical lymph nodes dissection. Pathological examination revealed multiple NET metastases (Figure 1A,B) positive for Syn and CgA and negative for TTF1, CDX2, CK19, CK7, and Ct (Figure 1C); MIB‐1 proliferative index was <1%; and a coexisting papillary thyroid carcinoma (0.7 cm) (Figure 1D–F). Massive lymph nodes metastasis was found. One month later, liver recurrence occurred. Treatment with somatostatin analogs and interferon achieved temporary disease control. One year later, widespread liver metastases were found. Chemotherapy (cisplatin + etoposide) resulted in partial response, but the disease progressed, leading to palliative care.

### 3.2 Case 2*

In 2018, a 61‐year‐old woman with a history of left ovarian strumal carcinoid (treated in 1988 and 1995),^11^ cerebral aneurysm, and hypertension was evaluated at Mauriziano Hospital (Turin) for an incidental right adnexal mass. Tumor markers (CA125, CA19.9, CEA), CgA, and 5‐hydroxy‐indole acetic acid (5HIAA) were found to be normal. The presence of a 3.4 cm right adnexal mass was confirmed by abdominal CT examination, which also showed two enlarged left para‐aortic lymph nodes of 2.2 cm each, suspicious for adenopathy without carcinomatosis or ascites. ^18^F‐fluorodeoxyglucose (FDG) PET‐CT scan showed mild uptake in the left para‐aortic region (SUV max 3.7). Given the radiological findings, peritoneal washing, right oophorectomy, and removal of bulky lymph nodes were carried out. On definitive histological report, according to World Health Organization WHO 2014 classification, the lesion was diagnosed as monoderm cystic teratoma of the ovary with an area of struma ovarii, including a focus of well‐differentiated NET (Syn+, CD56+, Neuron Specific Enolase (NSE)+, CDX2 focally +, CgA−, Thyroglobulin−, TTF1−). The proliferative index Ki 67 was <2%. Furthermore, of the 10 para‐aortic lymph nodes identified, only one was metastatic. Follow‐up with ^68^Gallium (Ga) DOTATOC PET‐CT showed uptake in the right side of the neck; thyroid ultrasound and FNA revealed a right lobe hypoechoic nodule with regular margins of 0.8 × 0.5 × 0.9 cm with peripheral vascularization, confirmed as NET metastasis. At immunocytochemistry, the tumor cells were positive for Syn, focally positive for galectin, TTF1, CDX2, thyroglobulin, and negative for Ct and CgA. Thyroid function was found to be normal (TSH 0.8 μUI/mL, Ct <2 pg/mL). After total thyroidectomy and central neck dissection, NET metastases were found in the thyroid and 3/5 lymph nodes (Ki 67 index <2%). She began Lanreotide therapy and levothyroxine. Later imaging revealed multiple liver metastases (the largest of 2.5 cm in the sixth segment), confirmed by biopsy. She remains on somatostatin analogs with close follow‐up.

*The clinical history before the appearce of the thyroid metastasis has been already published by Borghese et al.^11^

### 3.3 Case 3

**FIGURE 2:**
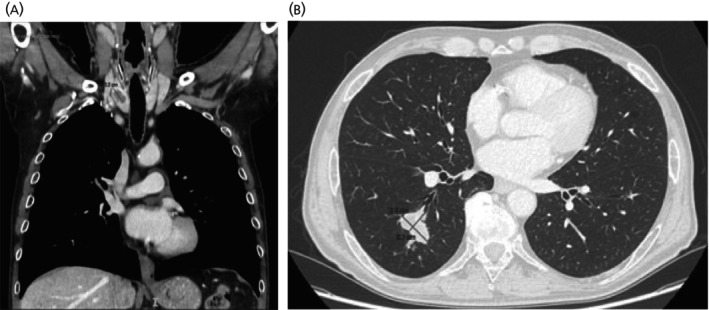
(A) CT scan of nodule with hypodense central component of the right thyroid lobe and (B) CT scan of hypodense lesion in the apical segment of the right lower pulmonary lobe.

In 2022, a 74‐year‐old Caucasian man with a history of type 2 diabetes mellitus, ischemic heart disease, prostate cancer, and hypertension was evaluated at the Endocrinology Department of the University Hospital of Ferrara for a thyroid nodule found incidentally during a Doppler ultrasound (US). Thyroid US revealed a 2.1 cm hypoechoic right lobe nodule with regular margins, internal macrocalcification, and peripheral vascularization. FNA showed poorly differentiated neoplastic cells, focally positive for CgA and Syn. Thyroid function was normal (TSH 0.73 μUI/mL, FT4 7.8 pg/mL, Ct <1 pg/mL) and tumor markers (CA19.9 and CEA) were normal, except for slightly elevated NSE (11.1 μg/L, with a normal range < 10.0 μg/L). The staging imaging study confirmed the presence of the thyroid nodule (Figure 2A) and, concomitantly, revealed the presence of a solid hypodense lesion with polylobed margins of 3.0 × 2.1 cm in the apical segment of the right lower pulmonary lobe (Figure 2B). Bronchoscopy and biopsy confirmed a small‐cell lung neuroendocrine carcinoma (Ki 67 90%), with immunohistochemistry matching the thyroid lesion (positive for CgA, Syn, CAM5.2 and INSM1 and negative for TTF1), indicating metastasis to the thyroid. 18F‐FDG PET‐CT showed high uptake in both lung (SUV max 13.9) and thyroid (SUV max 9.6) and moderate uptake in a right hilar lymph node. Unfortunately, data on treatment and follow‐up were not available.

## 4 LITERATURE REVIEW

The study search identified 27 studies, from 1983 to 2023, 25 case reports^12^, ^13^, ^14^, ^15^, ^16^, ^17^, ^18^, ^19^, ^20^, ^21^, ^22^, ^23^, ^24^, ^25^, ^26^, ^27^, ^28^, ^29^, ^30^, ^31^, ^32^, ^33^, ^34^, ^35^, ^36^ and two case series,^4^, ^37^ including six and two patients, respectively, accounting for a total number of 33 cases of thyroid metastases from NENs. Patients' ages ranged from 17 to 85 years with a mean age of 55.8 ± 14.2 years (14 males, 42.4%). The majority of cases (16/33, 48.5%) originated from a thoracic NEN^4^, ^16^, ^17^, ^18^, ^20^, ^21^, ^22^, ^27^, ^30^, ^31^, ^32^, ^37^; in 10 out of 33 cases (30.3%) the primary tumor was a GEP‐NEN^4^, ^12^, ^13^, ^14^, ^15^, ^19^, ^23^, ^25^, ^28^, ^33^; in three cases (9%) thyroid metastasis arose from a Merckel cell carcinoma^24^, ^26^, ^29^ and in the remaining cases, two originated from the cervix (6%),^34^, ^36^ one from the prostate (3%),^35^ and in one case the primary tumor site was unknown.^4^

Thyroid metastases were generally metachronous with a median time to diagnosis from the primary tumor of 48 months (range 1–252 months); in six out of 33 cases (18%) the diagnosis of thyroid metastases was synchronous with that of the primary tumor, and in three cases (9%) it was previous.

Details of case reports and series including patients with thyroid metastases from thoracic NENs are summarized in Table 2. The most frequent histology of the primary tumor was a well differentiated lung NEN (12/16 cases, 75%), specifically atypical carcinoid in three cases, typical in five cases, not specified in the remaining cases. In four cases a poorly differentiated histology was found (3 lung NEN and 1 thymoma). In seven cases out of 16 (43.7%) the diagnosis of thyroid metastasis was made by FNA, in four cases by histology (25%), in four other cases (25%) by a nuclear medicine imaging followed by FNA, and in one case (6.2%) by nuclear medicine followed by histology. In eight out of 12 cases (66.7%) for which the localization of metastasis has been reported, the thyroid lesion was in the right lobe. US features were described just in two cases and the nodules were hypoechoic, one of them with irregular margins. In three cases a multinodular goiter was described. The immunohistochemistry profile showed positivity for CgA in the great majority of cases (15/16, 93.7%), followed by Syn which was expressed in 9 (56.2%). In one case (6.2%) TTF1 was found positive. Tissue Ct was expressed just in one case (6.2%) in the presence of higher serum level of Ct (730 pg/mL <10) without a peak after pentagastrin infusion (758 pg/mL). In the majority of cases (14/16, 87.5%) surgery was the first therapeutic choice for the primary tumor followed by chemotherapy and radiation based on the final histology and staging. In 13 cases (81.2%), a total or partial thyroidectomy with lymphonodes dissection was performed for the treatment of thyroid metastasis. Finally, the prognosis was good in nine patients (69%) with a complete or partial response and poor in four patients (30.8%) because of a progressive disease; in the remaining cases it was not available.

**TABLE 2:** Details of case reports and series on thyroid metastasis (MTS) from thoracic neuroendocrine neoplasm (NEN).

Author, year	Age, sex	Site of thyroid MTS	US features	Site of primary tumor	Primary tumor size (cm)	Thyroid MTS size (cm)	MTS diagnosis (timing)	Primary histology, Ki 67, stage	IHC of thyroid MTS	Serum NE biomarkers	Treatment of primary tumor	Treatment of MTS	Follow‐up
Krausz et al., 1996^16^	36, F	Right lobe	Multiple solid nodules in both lobes	Left upper pulmonary lobe	NA	1.9	Octreoscan (after 15 years)	Bronchial carcinoid	CgA+	NA	Left upper lobectomy	NA	SSA for metastatic spread
Leboulleux et al. 1999^4^	40, M	Na	Multinodular goiter with supra‐clavicular limp nodes	Lung	5.0	NA	FNA	PD NEN	NSE+, CEA+, CgA−, Ct−	Increased NSE, Somatostatin, Ct Negative	Surgery‐Cisplatin+Etoposide for Metastatic Spread	NA	CR, alive after 19 months
	63, F	NA	Progressive increasing thyroid nodule without neck lymph nodes	Lung	3.5	1.0	FNA	WD NEN	NSE+, Syn+, CgA+, CEA+, Ct−	Increased CgA, Ct Negative	Surgery	Total thyroidectomy with cervical lymph node dissection SSA, 5‐fluorouracil and streptozocin for metastatic spread	PD, alive after 49 months
	67, M	NA	Fixed nodule with neck lymph nodes	Thymus	NA	NA	FNA (prior of primary)	PD Thymoma	NSE+, CgA+, CEA+, Ct−	Ct Negative	Cisplatin + etoposide	Total thyroidectomy with lateral tracheal resection and lymph node dissection	PD, dead after 15 months
	58, M	NA	Multinodular goiter with neck lymph nodes	Lung	15.0	NA	Surgery (after 2 years)	WD Bronchial NET	CEA+, Ct+, CgA+, Keratin+	Increased Ct (730 Pg/ml vn <10, Peak After PG 758 Pg/ml), CEA, NSE, Glycoprotein Human Alpha Subunit	Lung surgery and radiotherapy	Neck surgery, 5‐fluoro‐uracil, dacarbazine, doxorubicin and streptozocin	PR, dead after 52 months
Filosso et al. 2004^17^	55, F	Right lobe	NA	Right lower pulmonary lobe	7.0 × 3.0	2.0 × 1.0	Octreoscan + FNA (after 3 years)	Atypical bronchial carcinoid, Ki 67 6%, Pt4 N1	CgA+, Tg−, Ct−	CgA 197 Ng/ml (20–100), NSE 62 Ng/ml (<12.5)	Right pneumonectomy with systemic lymphadenectomy + octreotide 30 mg	Radical thyroidectomy and cervical lymphadenectomy	Patient alive and well after 13 months (octreotide 30 mg)
Maly et al. 2004^18^	42, F	Right lobe	NA	Right lower lobe of the lung	3.0	2.5	FNA (prior of the primary)	Typical bronchial carcinoid	NSE+, CgA+, Syn+, Ct−, CEA−	Normal Ct	Surgical resection of the right lower lobe and ipsilateral hilar lymph node sampling.	Total thyroidectomy and cervical lymph node dissection, chemotherapy	Alive after 28 months
Yamada et al. 2007^20^	68, F	Right lobe	NA	Right lower lobe of the lung	4.0	4.0	Histology (synchronous)	Large‐cell lung neuroendocrine carcinoma, Pt2N01	CD56+, Syn+, CgA+, CEA+, TTF1+, P53+, Ct−, Tg−	Increased CEA 62.4 ng/ml (<5), CYFRA 6.0 ng/ml (< 2.5), Normal Ct	Right lower lobectomy of the lung, and lymph node dissection radiotherapy	Right lobectomy of the thyroid	Alive after 6 years
Osawa et al. 2007^21^	71, M	Right lobe	NA	Right upper pulmonary lobe	NA	1.2 × 0.8	FNA (4 years) (isolated MTS)	Small and large‐cell lung neuroendocrine carcinoma, T2N2M0	CD56+, Cga+, Ct−, Tg−	Normal Ct	Right upper lobectomy+ adjuvant chemotherapy followed by prophylacticcranial irradiation	Metastasectomy and adjuvant chemotherapy including cisplatin and irinotecan	Disease free, alive after 16 months
La Rosa et al. 2009^22^	37, F	Right lobe	NA no lymph nodes enlargement	Lower lobe of the right lung (know from 8 years)	3.5	1.4	Octreoscan + FNA (synchronous)	Typical lung carcinoid	CgA+, Syn+, SSR2A+, Ct−, CEA−, Serotonin−, Somatostatin, GRP−, P53−	Normal Ct (42 Pg/ml <100)	Right lung lobectomy	Total thyroidectomy	Alive and disease free (7 years)
Sivrikoz et al. 2012^37^	17, F	Right lobe	NA	NA	NA	1.0	FNA	Atypical mediastinal carcinoid, Ki 67 10%	CgA+, Syn+, Ct−, CEA−, TTF1−	ACTH (Ectopic CS) Normal Ct And 5HIAA	Surgery + SSA	Total thyroidectomy, octreotide 30 mg	SD
	54, F	Right lobe	NA	Right lung	NA	3.0	FNA	Typical lung carcinoid	CgA+, Syn+, Ct−, CEA−, TTF1−	Normal Ct	Right pneumonectomy, thymectomy and lymph node dissection	Total thyroidectomy	NA
Koraitim et al. 2016^27^	68, M	Left lobe	NA	Lung	NA	2.0	Histology (after 7 years)	Bronchial Carcinoid	CgA+, Syn+, CD56+	NA	Lung lobectomy	Hemithyroidectomy	PD after 30 months
Albano et al. 2021^30^	62, F	Left lobe	Solid, hypoechoic with irregular margin	Left Lung	NA	NA	^68^Ga‐DOTATOC PET/CT + FNA	Atypical lung carcinoid, Ki 67 25%, Pt3n2m0	CgA+, Syn+	Normal Ct and CEA	Left pneumectomy with hilum‐mediastinal lymph node dissection	NA	NA
Ugolini et al. 2022^31^	70, M	Left lobe	NA	NA	NA	NA	Histology (after 13 years)	Typical Lung Carcinoid	CgA+	NA	NA	Total thyroidectomy	NA
Dello Spedale Venti et al. 2022^32^	40, M	Left lobe + right lobe	Solid, hypoechoic	Right upper pulmonary lobe	4.2	1.1 (right), 0.4 (left)	^68^Ga‐DOTATOC PET/CT + FNA (after 18 years)	Central typical bronchial carcinoid	Syn+, CgA+, NSE+, CD56+, CK AE1/AE3+, Tg−, Ct−, CEA−, TTF1−, High molecular weight cytokeratin−, CD10−, S100−	Normal Ct	Lung lobectomy with hilum‐mediastinal lymphadenectomy adjuvant chemotherapy and radiotherapy	Thyroidectomy	Disease free (suspected lymph node), alive after 7 months (data not published)

*Note*: Number of patients is in brackets.

Abbreviations: ACTH, adrenocorticotropic hormone; CD10, cluster of differentiation 10; CD56, cluster of differentiation 56; CEA, carcinoembryonic antigen; CgA, chromogranin A; CK AE1/AE3, cytokeratin AE1/AE3+; Ct, calcitonin; CYFRA, fragment of cytokeratin 19; CR, complete response; FNA, fine needle aspiration; GRP, gastrin releasing peptide; IHC, immunohistochemistry; NA, not available; NSE, neuron specific enolase; p53, tumor suppressor protein; SSA, somatostatin analogues; SSR2A, somatostatin receptor subtype 2A; Syn, synaptophysin; S100, small calcium binding proteins; PD, progressive disease; PET/CT, positron emission tomography‐computed tomography; PR, partial response; Tg, Thyroglobulin; TTF1, transcriptional thyroid factor 1; US, ultrasound; WD, well differentiated.

Details of case reports and series including patients with thyroid metastases from GEP‐NENs are summarized in Table 3. The most frequent primary site of the tumor was the intestine (7/10 cases, 70%), at different levels (ileum, rectum, caecum and appendix), followed by the pancreas (3/10 cases, 30%). The most frequent histology was a well‐differentiated neoplasm (8/10 cases, 80%), but grading was specified only in two cases: one G2 tumor with a Ki 67 of 5–10% and one G1 tumor with Ki 67 of 1%. In four cases out of 10 (40%) the diagnosis of thyroid metastasis was made by FNA, in four cases (40%) by histology (2 autopsy findings), and in two cases (20%) by a nuclear medicine imaging followed by FNA. The localization of thyroid metastases has been reported to be in the right or left lobe indifferently. US features were described in just two cases (20%), in one the nodule was well defined and hypoechoic, and in the other case a multinodular goiter was found with two prominent nodules described as isoechoic, regular, and uneven halo around the edge, with poor blood flow. Serum Ct was found elevated only in two cases (20%), in one ileal NET with a slight increase and in one pancreatic NET with an increase of 40 fold; unfortunately, immunohistochemistry was not available for these two tumors.

**TABLE 3:** Details of case reports on thyroid metastasis (MTS) from gastroenteropancreatic (GEP)‐ neuroendocrine neoplasm (NEN).

Author, year	Age, sex	Site of thyroid MTS	US features	Site of primary tumor	Primary tumor (cm)	Thyroid MTS size (cm)	MTS diagnosis (timing)	Histology, Ki 67, stage	IHC of thyroid MTS	Serum NE biomarkers	Treatment of primary tumor	Treatment of thyroid MTS	Follow‐up
*Gastric‐NEN*													
Poiana et al., 2011^25^	70, F	Left lobe + Isthmus	NA	Stomach	5	NA	Thyroid surgery (after 2 years)	Poorly differentiated carcinoma (with small cells), Ki 67 25%, T3N1M1	Syn+, Cga+, Cytokeratin AE1–AE3+, CD56+, Ct−, TTF1−, Tg−, CD3−, CD20−, CD138−, S100−, CK20− No lymph nodes	Increased CgA, Serotonin, NSE, 5‐HIAA	Surgery, OCT LAR 20 mg	NA	Alive without other metastases
*Pancreatic‐NEN*													
Vorne et al., 1990^14^	54, M	Right lobe	NA	Pancreas	8	1.5	^201^TI‐^99^TC scintigraphy (synchronous)	Carcinoid tumor (autopsy findings)	NA	Ct 1200 pmol/L (<30), 5‐HIAA normal	None	NA	Dead after 6 months
Leboulleux et al., 1999^4^	58, M	NA	Multinodular goiter with neck lymph nodes	Pancreas in MEN 1	3	NA	Cytology (synchronous)	Well‐differentiated	NA	Increased NSE, CgA, Ct negative	Doxorubicin and streptozocin for metastatic spread (bone)	NA	Alive after 6 months
Massani et al., 2010^23^	65, M	Right lobe	Well‐defined hypoechoic	Pancreas	<3	3	FNA + Thyroid surgery (prior of the primary) (isolated MTS)	High grade small cell endocrine carcinoma	NA	Normal Ct	Total pancreaticoduodenectomy and chemotherapy carboplatino+etoposide	Right lobectomy	PD (bone), alive after 18 months
*Intestinal‐NEN*													
Marks et al., 1983^13^	52, F	Left lobe	NA	Ileum of Meckel's diverticulum	2	2	Scintigraphy + FNA	Carcinoid tumor	NA	Elevated Ct (0.4 ng/ml, <0.2 + − 0.1), 5‐HIAA (23 mg/24 < 10 mg/24).	Surgery	NA	Dead for surgical complications
Vija Racaru et al., 2019^28^	58, F	Isthmus	NA	Ileum	NA	NA	^68^Ga‐DOTATOC PET/CT + FNA (after 5 years)	Metastatic intestinal neuroendocrine tumor (G1, Ki 67 1%)	CD56+, CgA+, Syn+, Ct−	Ct Negative	Surgery and liver embolization	Radioligand Therapy 177Lu‐DOTATATE	NA
Mattavelli et al., 2008^12^	41, F	Right lobe	NA	Appendix	0.5	3	FNA (after 21 years)	Well‐differentiated, low‐grade neuroendocrine carcinoma	Keratins+, CgA+, Cgb+, Ct−, TTF1−	NA	Surgery and chemotherapy	Right hemithyroidectomy plus, isthmectomy en bloc with the prethyroid muscles	Alive after 18 months
Papi et al., 2005^19^	56, F	Left lobe	NA	Colon (Caecum)	NA	NA	Thyroid surgery	Well‐differentiated	Keratins+, CgA+, Syn+, NSE+, Serotonin+, CDX2+, Ct−	NA	Surgery	NA	PD (bone), alive after 18 months
Lertprasertsuke et al., 1990^15^	58, M	NA	NA	Rectum intestine	NA	Multiple nodules (3–8 mm)	Autopsy (multiple metastases: liver, pancreas)	Rectal carcinoid	NSE+, Keratins+, Somatostatin+, PP+, PAP+, Ct−, CEA−, CgA−, Tg−	5HIAA, histamine, ACTH, epinephrine, norepinephrine and dopamine normal	Explorative laparotomy	NA	Dead after 3 months
Zhang et al. 2023^33^	56, F	Multiple nodules	Isoechoic, regular shape, and an uneven halo around the edge, with poor blood flow	Rectum	3.1 × 2.5	2.0 × 2.0 cm (right) and 1.0 × 1.5 cm (Left)	FNA (synchronous, no other MTS)	Rectal neuroendocrine tumor, G2 Ki 67 5%–10%, Stage IIIB	CK+, Tg−, TTF1 Slightly Scattered, HBME‐1−, S‐100−, Ct−, CgA+, Syn+, Ki‐6710‐20%, CK19 +, CK20−, PAX 8−, CEA−, CDX2− and SATB2−	NA	Radical rectal resection + cisplatin and etoposide	Bilateral total thyroidectomy + bilateral central lymph node dissection	PD (liver), dead

*Note*: Number of patients is in brackets.

Abbreviations: 5HIAA, 5‐hydroxy‐indole acetic acid; CD3, cluster of differentiation 3; CD20, cluster of differentiation 20; CD56, cluster of differentiation 56; CD138, cluster of differentiation 138; CDX2, caudal‐related homeobox transcription factor 2; CEA, carcinoembryonic antigen; CgA, chromogranin A; CgB, chromogranin B; CK20, cytokeratin 20; Ct, calcitonin; FNA, fine needle aspiration; G1, grade 1; IHC, Immunohistochemistry; NA, not available; NET, neuroendocrine tumor; NSE, neuron specific enolase; PAP, prostatic acid phosphatase; PD, progressive disease; PET/CT, positron emission tomography‐computed tomography; PP, pancreatic polypeptide; Syn, synaptophysin; S100, small calcium binding proteins; TC, technetium; Tg, Thyroglobulin; TI, Thallium; TTF1, transcriptional thyroid factor 1; US, ultrasound.

The immunohistochemistry profile, available in six cases (60%), showed positivity for CgA in the great majority of them (5/6, 83.3%), followed by Syn and keratins, which were expressed in 4 (66.6%). When reported, Ct was negative and TTF1 was also negative or slightly scattered. In the majority of cases (9/10, 90%) surgery was the first therapeutic choice for the primary tumor, followed by chemotherapy in three cases and somatostatin analogs in one case. Only in three cases was a total or partial thyroidectomy with lymphonodes dissection performed for the treatment of thyroid metastasis. Finally, despite the well‐differentiated histology, the prognosis was poor in six patients (66.6%) because of progressive disease or death, good only in three patients alive at last follow‐up; in the remaining case, it was not available.

Details of case reports including patients with thyroid metastases from rare sites of neuroendocrine carcinomas (NEC) are summarized in Table 4, including three Merckel cell carcinomas, two uterine cervix NECs, of which one mixed adenoneuoroendocrinecarcinoma (MANEC), and one prostatic large cell NEC. In four cases out of 6 (66.6%) the diagnosis of thyroid metastasis was made by ^18^F‐FDG‐PET‐CT and in the remaining cases the diagnosis was histological. Thyroid metastases have been reported in the right lobe in three cases (50%). US features were described just in three cases and the nodules were hypoechoic with irregular margins. The immunohistochemistry profile was variable according to the primary tumor, CgA, Syn, and keratins were generally positive, while Tg, Ct, and TTF1 were negative.

**TABLE 4:** Details of case reports on thyroid metastasis from rare sites of neuroendocrine carcinomas (skin, cervix and prostate).

Author, year	Age, sex	Site of thyroid MTS	US features	Site of primary tumor	Primary tumor size (cm)	Thyroid MTS size (cm)	MTS diagnosis (timing)	Histology, Ki 67, Stage	IHC Of thyroid MTS	Serum NE biomarkers	Treatment of primary tumor	Treatment of thyroid MTS	Follow‐up
Stoll et al.,^24^	50, M	Right lobe	Solid, hypoechoic, increased vascularity and irregular margins + two cystic nodules	Left distal forearm	NA	2.1	^18^F‐FDG‐PET/CT + FNA (after 4 years)	Poorly differentiated merkel cell carcinoma	NA	NA	Surgery + radiotherapy + chemotherapy	NA	NA
Tsoukalas et al. 2014^26^	73, F	Right lobe (submerged goiter)	Increase of the size of the submerged right lobe	Adipose tissue of the right inguinal area	4.6	NA	Histology (after 10 months)	Poorly differentiated small cell carcinoma, with histopathologic features of a merkel cell carcinoma	CAM 5.2+, CK20+, NF+, Cga+, Syn+, CD56+, TTF1−	Increased NSE 26 ng/ml (<16.3) And CgA 9.2 ng/ml (<5.6)	Surgery, chemotherapy, radiotherapy	Right lobectomy for compressive symptoms	CR
Vaiciunaite et al. 2019^29^	85, F	Isthmus	NA	Right posterior calf	1.0	2.0	^18^F‐FDG‐PET/CT (after 4 months)	Poorly differentiated merkel cell carcinoma T2N2M0, Stage IIIA	NA	NA	Surgery	Immunotherapy (multimetastatic disease)	Dead after 2 months
Ortiz et al. 2022^34^	48, F	Both lobes	Multinodular goiter right lobe 2.3 cm, left lobe 4.5 cm	Uterine cervix	NA	Both thyroid lobes ranging from 0.1 to 0.5	^18^F‐FDG‐PET/CT, FNA (misdiagnosis PTC), histology (after 1 month)	Uterine cervix NEC Stage 3B	CD56+, CK7 +, CK20 +, P16 +, Syn +; ER and CEA partially +; CK5/6−, TTF‐1−, and P63−	NA	NA	Total thyroidectomy for compressive symptoms	NA
Li Shua et al. 2022^36^	54, F	Left Lobe	Hypoechoic nodule with irregular shape, unclear boundary, around blood flow signals in color doppler flow imaging, and no lymphadenopathy	Uterine cervix	NA	1.1	Core needle biopsy, ^18^F‐FDG‐PET/CT (after 5 years)	Mixed adenoneuroendocrine carcinoma, stage IB	P16+, Syn+, CEA+, CgA+, CK+, and weakly positive for TTF1 Ki 67 70%	NA	Radical hysterectomy	Left hemithyroidectomy plus isthmectomy + chemotherapy	PD, dead after 1 year
Karvounis et al.^35^	78, M	Right Lobe	Two hypoechoic nodules with enlarged neck lymph nodes	Prostate	NA	NA	^18^F‐FDG‐PET/CT + FNA (synchronous)	De novo prostatic large‐cell neuroendocrine carcinoma	AR + Syn + CAM5.2+, PSAP+, CK8.18+, AMACR partially +, Tg−, Ct−, TTF‐1−, PSA−, CD56−, NSE−, and Keratin‐903—Ki 67 and CgA 30%	NA	Androgen deprivation therapy + chemotherapy	Palliative thyroidectomy and lymph nodes dissection	PD, dead after 4 months

Abbreviations: AMACR, alpha‐methylacyl‐CoA racemase; AR, androgen receptor; CAM 5.2, pancytokeratin; CD56, cluster of differentiation 56; CgA, chromogranin A; CK 20, cytokeratin 20; FNA, fine needle aspiration; IHC, immunohistochemistry; NA, not available; NF, neurofilaments; NSE, neuron specific enolase; Syn, synaptophysin; TTF1, thyroid transcription factor 1; PD, progressive disease; PET/CT, positron emission tomography‐computed tomography; PSAP, prostate‐specific acid phosphatase; PTC papillary thyroid carcinoma; US, ultrasound.

## 5 DISCUSSION

The thyroid gland is an uncommon site of metastatic disease, accounting metastases for 1.4%–3% of thyroid tumors, likely because of peculiar features of glandular microneviroment such as a fast arterial blood flow and high concentrations of oxygen and iodine, that might prevent the anchorage and subsequent growth of circulating tumor cells.^3^ However, in autopsy studies thyroid metastases are more frequent, accounting 1.9%–24.2% in patients who died because of other cancers.^12^ Generally, metastases to thyroid originate from renal cell (48.1%), colorectal (10.4%), lung (8.3%), breast carcinoma (7.8%), and sarcoma (4.0%).^38^ However, even though it occurs very rarely (1.0%–4% of cases), the possibility of a metastasis from NENs should also be taken into account.^2^ Given their heterogeneity, this group of neoplasms has different behaviour, from silent to more aggressive forms that can lead to an incidental or late diagnosis, often only after the onset of distant metastases, found in more than 40% of patients at the time of diagnosis.^39^ Generally, well‐differentiated NENs, the so called NETs, are characterised by slow progression and good prognosis with long‐term survival. In this context, clinicians should suspect thyroid metastases from NEN in patients who present with a new thyroid nodule and a history of prior NEN, even if many years from the first diagnosis have passed. Metastatic NENs to the thyroid can also mimic a primary NET of the thyroid, in the great majority of cases MTCs.

The current literature review identified 33 cases of thyroid metastases from different kinds of NENs and, additionally, the case series derived from three Italian referral centers retrieved three more cases, reflecting the great biological and clinical heterogeneity of this group of neoplasms. Thyroid metastases from NENs are most common in women than in men and in the six decades of life. Overall, in the majority of cases (48.8%) the primitive tumor was a lung NEN, mostly well‐differentiated (75%), followed by a GEP‐NEN, accounting for almost 30.3% of cases. Other less frequent sites of origin are skin, with three cases of Merkel cell carcinomas, and the genitourinary tract. In females, the cervix is the most frequent site of NEN, but only two cases of NEC of the uterine cervix with metastases to the thyroid gland were reported in the literature.^34^, ^36^ In males, de novo prostatic NEC can metastasize almost anywhere in the body, including the thyroid and adrenal glands.^35^ Time to thyroid metastasis from the diagnosis of NEN was extremely wide, ranging from a few months to dozens of years, sometimes being the diagnosis of thyroid metastasis concurrent with or previous to that of the primary tumor. Although they have been described just in a subset of patients, US features of NEN metastases seem to be superimposable to those of differentiated thyroid carcinoma: hypoechoic nodules with irregular margins. Moreover, as for differentiated thyroid carcinoma, FNA was the first step in the diagnostic work‐up, with only one case of misdiagnosis as a papillary thyroid carcinoma^34^; conversely, Chung et al. reported a false‐negative rate of 28.7% for FNA in the diagnosis of metastatic thyroid tumors.^38^ Only in one case was a core needle biopsy preferred to obtain more adequate tissue samples for histopathological and immunohistochemical diagnosis and to reduce the risk of false‐negative diagnosis.^36^

Since MTC shares morphological aspects with other thyroid NENs, including metastases, it may be very difficult to differentiate MTC from a metastatic nodule of a NEN on histological exam; thus, the immunohistochemistry is essential for differential diagnosis, playing a positive medical history of previous NEN a pivotal role. Similarly, NEN metastases to the thyroid gland can also share histological features as that of thyroid paraganglioma. The clinical history, the presence of a predominantly interstitial pattern of spread, with multiple tumor foci, peculiar morphological aspects as tumor cells organized in subepithelial ball‐like collections and rosette formations with lumen, together with the lack of immunoreactivity for Ct and CEA favor the diagnosis of NEN metastases.^10^ Data from the current literature review and case series show that, in almost all cases, the immunohistochemistry was positive for CgA and Syn, which are generic neuroendocrine markers; but, concomitantly, it was negative for Ct and TTF1, usually expressed by MTC.

Nuclear medicine imaging, particularly Ga‐peptides PET‐CT in well‐differentiated NEN, has also an important role in the differential diagnosis work‐up, pointing out the neuroendocrine fingerprint of the thyroid nodule and helping disease staging by identifyng other metastatic lesion and, in some cases, the primitive tumor.^40^ Conversely, ^18^F‐FDG PET‐CT was the first diagnostic modality for thyroid metastases from NEC, that were detected in the staging of the primary disease. In these cases, US exam can be useful to malignancy risk stratification of thyroid incidentalomas to select that nodules to submit to subsequent diagnostic work‐up.^41^ Serum neuroendocrine biomarkers can rarely help in diagnostic process, only a slight increase in CgA and NSE was reported in seven cases, and in one case an ectopic Cushing syndrome was described. To note, serum Ct was found increased in three cases, one from a lung well‐differentiated NEN and two from GEP‐NEN (ileum and pancreas). Serum Ct is usually used in the diagnosis and follow‐up of MTC, but it can be find increased also in Ct‐secreting NEN, complicating the differential diagnostic work‐up in presence of thyroid nodules and NEN.^5^, ^6^, ^7^, ^8^ In such cases clinicians should take into account the complete patient's medical history, the hormonal assessment, potentially including stimulation test for Ct, the integration of conventional and nuclear imaging, that can help to ensure accurate diagnosis and appropriate clinical management. Although rare, a sospicious thyroid nodule in a patient with a medical history of previous or concurrent NEN should be considered metastatic until proven otherwise. However, sometimes, only histology from partial or total thyroidectomy allow to reach a definitive diagnosis.

The treatment strategy varied among the studies from surgery to chemotherapy according to the heterogeneity of the primary tumor, the grade and stage of the neoplasm, and the patient's general condition. A preoperative diagnosis of metastatic disease could avoid unnecessary thyroid surgery, as patients usually have multiple metastases and need systemic treatment after a multidisciplinary evaluation. However, in selected patients with isolated thyroid metastasis, the surgical management should be considered to obtain curative resection or prevent tumore recurrence, or in cases with compressive symptmos in order to avoid important morbidity due to mass effect in the neck.^23^, ^42^ In thyroid metastases from other cancer subtotal or total thyroidectomy did not affect patients survival,^43^ therefore, it reasabable that a personalized surgical strategy should be considered in each case. In this review, prognosis generally depends on primary site of origin, differentiation—well or poorly differentiated NEN—and the presence of isolated or multiple metastatic disease. Solitary thyroid nodule as a primary presentation of a metastatic disease was associated with a good prognosis expecially if the primary site of origin was thoracic, conversely the prognosis was generally poor in patients affected by a GEP‐NEN.

## 6 CONCLUSIONS

Thyroid metastases from NEN are a very rare entity; anyway, they have to be taken into account in the diagnostic work‐up of suspicious thyroid nodules in patients with a positive medical history of previous NEN, especially thoracic NEN, even after many years from initial diagnosis. Differential diagnosis from primary intrathyroidal NEN could be challenging, particularly in presence of isolated metastatic disease. Thyroid metastases from NEN share US features of differentiated thyroid carcinoma, being in the majority of cases hypoechoic with irregular margins, and histological aspects of MTC and NET of the thyroid, such as paraganglioma and CNNET, thus advocating immunohistochemistry as the key diagnostic tool for their identification. A prompt and right diagnosis, integrating US, cito‐histology, and nuclear medicine is mandatory because it is crucial to plan an appropriate therapeutic strategy and for prognostic implications. In conclusion, if you hear hoof beats behind you, do not look back always expecting to see a horse; sometimes, expect a “polka‐dotted” zebra.

## AUTHOR CONTRIBUTIONS

**Tiziana Feola:** Writing – original draft; conceptualization; methodology. **Alessia Cozzolino:** Conceptualization; writing – original draft. **Federica Grillo:** Conceptualization; writing – review and editing; investigation. **Maria Francesca Birtolo:** Conceptualization; writing – review and editing. **Irene Aini:** Conceptualization; writing – review and editing. **Erika Messina:** Conceptualization; writing – review and editing. **Roberto Minotta:** Conceptualization; writing – review and editing. **Alessia Filice:** Visualization; writing – review and editing. **Isabella Zanata:** Writing – review and editing; visualization; investigation. **Paola Razzore:** Writing – review and editing; conceptualization; investigation. **Manila Rubino:** Writing – review and editing; visualization. **Andrea M. Isidori:** Supervision; writing – review and editing. **Annamaria Colao:** Writing – review and editing; supervision. **Antongiulio Faggiano:** Conceptualization; writing – review and editing; supervision. **Elisa Giannetta:** Conceptualization; writing – original draft; supervision.

## FUNDING INFORMATION

This research was supported by co‐funding of the European Union—Next Generation EU, Mission 4 Component 2 Investment 1.5, project Rome Technopole—code ECS 00000024 (CUP: B83C22002820006).

## CONFLICT OF INTEREST STATEMENT

Andrea M. Isidori has been a consultant for Novartis, Takeda, Recordati, and Sandoz companies and has received unconditional research grants from Shire, IPSEN, and Pfizer. All the other authors have nothing to disclose.

## PEER REVIEW

The peer review history for this article is available at https://www.webofscience.com/api/gateway/wos/peer‐review/10.1111/jne.70061.

## Data Availability

Data sharing is not applicable to this article as no new data were created or analyzed in this study.
